# N_2_-fixing tropical legume evolution: a contributor to enhanced weathering through the Cenozoic?

**DOI:** 10.1098/rspb.2017.0370

**Published:** 2017-08-16

**Authors:** Dimitar Z. Epihov, Sarah A. Batterman, Lars O. Hedin, Jonathan R. Leake, Lisa M. Smith, David J. Beerling

**Affiliations:** 1 Department of Animal and Plant Sciences, University of Sheffield, Sheffield S10 2TN, UK; 2 School of Geography and Priestley International Centre for Climate, University of Leeds, Leeds LS2 9JT, UK; 3 Smithsonian Tropical Research Institute, Balboa, Ancon, Panama; 4 Department of Ecology and Evolutionary Biology, Princeton University, Princeton, NJ 08544, USA

**Keywords:** tropical forests, N_2_-fixation, legume trees, rock weathering, CO_2_ sequestration, Cenozoic

## Abstract

Fossil and phylogenetic evidence indicates legume-rich modern tropical forests replaced Late Cretaceous palm-dominated tropical forests across four continents during the early Cenozoic (58–42 Ma). Tropical legume trees can transform ecosystems via their ability to fix dinitrogen (N_2_) and higher leaf N compared with non-legumes (35–65%), but it is unclear how their evolutionary rise contributed to silicate weathering, the long-term sink for atmospheric carbon dioxide (CO_2_). Here we hypothesize that the increasing abundance of N_2_-fixing legumes in tropical forests amplified silicate weathering rates by increased input of fixed nitrogen (N) to terrestrial ecosystems via interrelated mechanisms including increasing microbial respiration and soil acidification, and stimulating forest net primary productivity. We suggest the high CO_2_ early Cenozoic atmosphere further amplified legume weathering. Evolution of legumes with high weathering rates was probably driven by their high demand for phosphorus and micronutrients required for N_2_-fixation and nodule formation.

## 1. Introduction

Biogeochemical weathering of silicate rocks (e.g. basalt, andesite, dunite) is a key process in the carbon cycle that acts as a long-term sink of atmospheric carbon dioxide (CO_2_) [1]. Consumption of CO_2_ by weathering is small (0.10–0.12 Gt C yr^−1^) on an annual basis [2] compared with carbon transfers in photosynthesis or respiration. However, net CO_2_ consumption by weathering is the dominant sink in the global carbon balance thus controlling atmospheric CO_2_ and climate patterns at scales of millennia or longer [2].

Numerous field studies have shown that plants accelerate rock weathering through a suite of increasingly well understood processes [3] (electronic supplementary material, figure S1). By increasing the soil pools of H^+^ ions, carbonic (H_2_CO_3_, from plant or soil respiration) and chelating organic (RCOO^−^) acids, plants and their symbiotic partners cause the weathering release of base cations (electronic supplementary material, figure S1) that ultimately lead to the formation of marine carbonates on the seafloor [2]. The rise of the first forests during the Devonian (419–359 Ma) [4] probably accelerated silicate weathering, contributing to the drawdown of atmospheric CO_2_ and establishing the basic features of the modern land carbon cycle. Today, forests are thought to enhance rock weathering by a factor of 2–10 compared with unvegetated catchments [5].

During the Cenozoic (past 65 Ma), the global biome transformation from palm-dominated Late Cretaceous forests to the highly productive and carbon-rich tropical forests that exist today, discussed in more detail in the next section, included the rise of trees in the ecologically important legume family (Leguminosae, or ‘legumes’). Legumes dominate large areas of modern tropical forests in both total number of tree species and in abundance within local forests [6].

Four lines of evidence suggest that the evolution of the dinitrogen (N_2_)-fixing rhizobial symbiosis (in which dinitrogen-fixing rhizobial bacteria are housed within specialized root nodules [7]) occurred as legumes radiated and spread in the early Cenozoic [9]. First, a whole-genome duplication event in the Papilionoideae clade, molecularly dated to 58 Ma, probably created the gene copies necessary for nodulation and N_2_-fixation to evolve [10]. Second, many modern rainforest -fixing legume trees are nodulated by β-rhizobia in the *Burkholderia* group [11]. Horizontal transfer of symbiotic *nod* genes between α-rhizobia and South American *Burkholderia* is dated to 60–50 Ma [12], indicating that compatible N_2_-fixing host trees may have appeared at that time. Third, the presence of fossil legume genera recovered from early Cenozoic deposits with present-day relatives capable of N_2_-fixation also supports the view that this capacity was developed in early members of the family, with our synthesis indicating that the majority of fossil taxa identified at the genus level of Palaeocene and Eocene age belong to N_2_-fixing genera (25 taxa) relative to non-fixing (16 taxa; electronic supplementary material, figure S2). Fourth, an increased proportion of legume fossil leaves recovered from 56 Ma old strata correlate with intensification of insect damage. This is a pattern consistent with the influx of fresh, fixed nitrogen (N) into the ecosystem [13].

Fossil genera, the symbiotic status of their nearest living relatives (electronic supplementary material, figure S2), evidence of increased insect damage in the fossil record in likely response to high foliar N and molecular clock dating therefore appear to indicate that N_2_-fixation and diverse mycorrhizal symbioses had evolved in legumes by the early Cenozoic.

Here, we review the rise of N_2_-fixing legume-rich tropical forests early in the Cenozoic and propose a new testable hypothesis for how the evolution of this biome may have strengthened the long-term carbon cycle feedbacks that helped shape Earth's CO_2_ and climate history in the Cenozoic.

## 2. Global rise of nitrogen-fixing legume-rich tropical forests

Late Cretaceous tropical floras were dominated by widely distributed palm communities from Africa to South America, a floristic region known as the Palmae Province [14–16]. Communities in both the Palaeo- and Neotropics contained abundant palms, including those resembling extant *Nypa* palms and suggestive of coastal intertidal habitats similar to mangrove forests, while other areas harboured palm-dominated dry forest communities. Unlike modern tropical forests, both of these communities were deprived of abundant dicot arboreal flora [14,15]. In Africa, leaf fossil and pollen evidence indicate that the dominant palm lineages began to decline around the Cretaceous–Palaeogene boundary [16] and completely disappeared in the fossil record during the Miocene [15]. Similarly, palm abundance in Neotropical areas decreased in the early Cenozoic, although palms remain an important element of these forests today [17]. The Palmae Province was replaced in Africa and assimilated in South America by the rise of modern tropical forests during the early Cenozoic. The earliest record of modern Neotropical forests—found in Colombia and dated to the Late Palaeocene (58 Ma)—indicates that the flora resembled the current day composition of plant families with abundant fossilized dicot and palm leaves, including numerous legumes [18]. Pollen records from Africa similarly show the rise of modern families of dicot trees following the Palaeocene [15,16].

**Figure 1.**
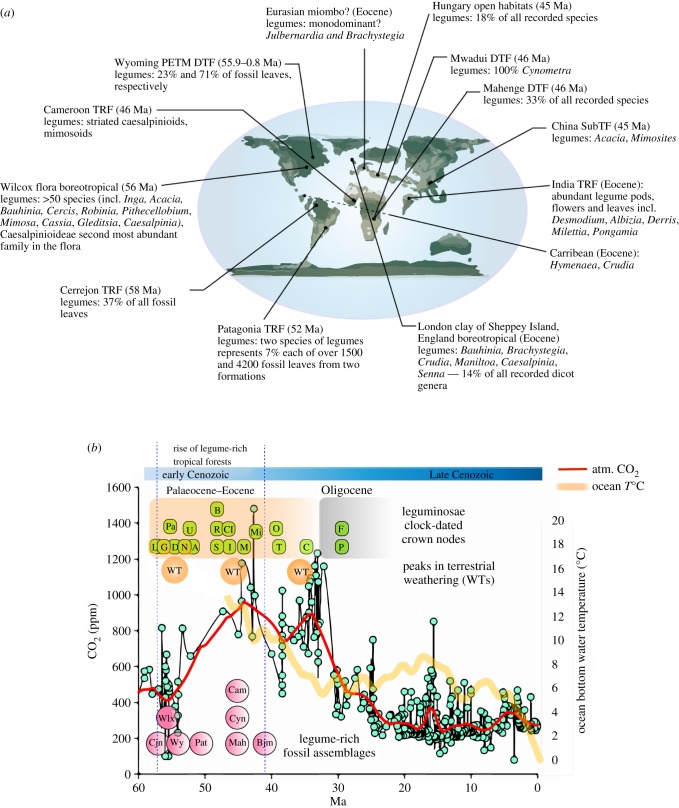
Global rise of legume-rich tropical forests during the early Cenozoic (58–42 Ma). (*a*) Global map of the major legume fossil records plotted on the Eocene continental configuration. Lines and their ball ends point to approximate locations. Caesalpinioids in the Wilcox flora are according the old pre-molecular taxonomy with a family status. DTF, dry tropical forest; SubTF, sub-tropical forest; TRF, tropical rainforest, boreotropical or BTF, a forest with mixed tropical and temperate species which is sometimes referred to as boreotropical. (*b*) Summary of the notable legume-rich fossil assemblages and all major molecular clock-dated crown nodes in the Leguminosae marking the rise of the legume-rich forests in the Palaeocene–Eocene plotted against atmospheric CO_2_ records (light blue dots and red Loess curve) using data from [19] and ocean bottom water temperature (orange semi-transparent curve) using data from [21]. Peaks in terrestrial weathering (WTs = 55, 48, 35 Ma) are estimated as levels of lateritization and bauxitization in [20]. Cjn, Cerrejon rainforest formation; Wlx, Wilcox boreotropical flora; Wy, Wyoming flora; Pat, Patagonia dry forests; Mah, Mahenge dry tropical forest; Cyn, *Cynometra*-monodominant stands in Mwadui; Cam, Cameroon tropical rainforest; Bjm, putative *Brachystegia*-*Julbernardia* miombo (macrofossils but not assemblage). Crown nodes include the divergence of L, Leguminosae; Pa, Papilionoideae; G, Genistoids; D, Dalbergioids; N, *Senna* clade; U, *Umtiza* clade; A, Amherstieae tribe (contains the majority EM taxa) after [23]; S, *Swartzia* clade; R, Robinioids; B, Mirbelioids; I, Indigoferoids; Cl, *Cladrastis* clade; M, Millettioids; Mi, Mimosoideae; O, *Peltophorum* clade; T, *Trifolium* (IRLC) clade; C, *Cercis* clade; P, *Poeppigia* clade; F, Fossil not supported *Brachystegia* clade (because fossils of *Brachystegia* and *Julbernardia* found much earlier and new estimates show that this divergence occurred 52.1 Ma—here marked as clade Amherstieae). Clock data references: all clade ages unless otherwise stated are after [9].

Pollen and leaf macrofossils indicate that legume taxa have comprised a key component of tropical forests since the early Cenozoic (figure 1*a*; electronic supplementary material, figure S2). While it is difficult to translate a taxon's abundance in the fossil record to abundance in a forest, the persistent recovery of legume pollen, leaves, flowers, fruits and wood indicate that legume trees were present and widespread in the flora of the Americas and Africa. The following observations can be drawn from early Cenozoic records: (i) legume leaves made up 21–73% of all fossilized leaves in South and North American forest assemblages [18,24]; (ii) legumes comprised 14–33% of all recorded taxa across tropical forests (figure 1) [25–27]; (iii) single legume tree species represented up to 7% of all fossil leaves (greater than 200 leaves) in species-diverse South American dry forests [28,29]; (iv) one legume tree species (the non-fixing *Cynometra*) formed a monodominant forest in Africa 46 Ma [26], with further monodominance indicated by the presence of Eocene fossils that belong to modern monodominant genera such as the non-fixing *Brachystegia* and *Julbernardia* (Eurasian deposits) and the non-fixing *Peltogyne* (South American formations) (electronic supplementary material, figure S2); (v) rainforests with abundant presence of caesalpinioid and mimosoid (many modern representatives of which are N_2_-fixing [7]) legumes were recorded in central Africa [15]; and (vi) tropical and temperate N_2_-fixing legume trees may have coexisted during warm Eocene climates in higher latitude boreotropical forests (England, Hungary, North America) [30].

Fossil evidence, therefore, indicates that early Cenozoic tropical forests (wet, dry and boreotropical) had evolved abundant legumes across continents (figure 1*a*). The timing of the early Cenozoic assembly of legume-rich tropical forests (58–42 Ma) as documented by the fossil record is similar to the molecular clock-dated diversification events in the legume clade (figure 1*b*; for recent changes in legume taxonomy, see [31]). Beneath these emerging tropical forests were substantial areas of unweathered rocks in tropical India [32], in South America, including the southeast part of the Amazon basin, and in the Amazon deltaic area [22] coinciding with peaks in terrestrial weathering (figure 1*b*) as evident from the recovery of highly weathered palaeosols [20].

## 3. Mechanisms of N_2_-fixing legume-driven enhanced weathering

Here, we propose that the rise of N_2_-fixing legume trees enhanced weathering through a series of processes associated with three abilities especially well developed in this group of trees: (i) to fix atmospheric N_2_, (ii) to build disproportionately N-rich leaf tissue, and (iii) to stimulate the primary production in ecosystems by redistributing fixed N to the soil and to neighbouring trees.

First, N_2_-fixing legumes have the ability to fix N at high rates in natural ecosystems [33]. Over time, fixers bring in substantial quantities of N and can provide the largest natural source of new N to ecosystems [34]. Soil N is high and nitrate and denitrification losses large (exceeding or rivaling many temperate forests exposed to N deposition) in tropical forests that harbour N fixers [33]. In a survey across 55 tropical forests, these systems naturally sustained loss rates of 4–6 kg N ha^−1^ nitrate, 6–10 kg N ha^−1^ of total dissolved N and 4–5 kg N denitrified; when corrected for low levels of atmospheric N deposition, these rates could only be explained by fixation [35].

**Figure 2.**
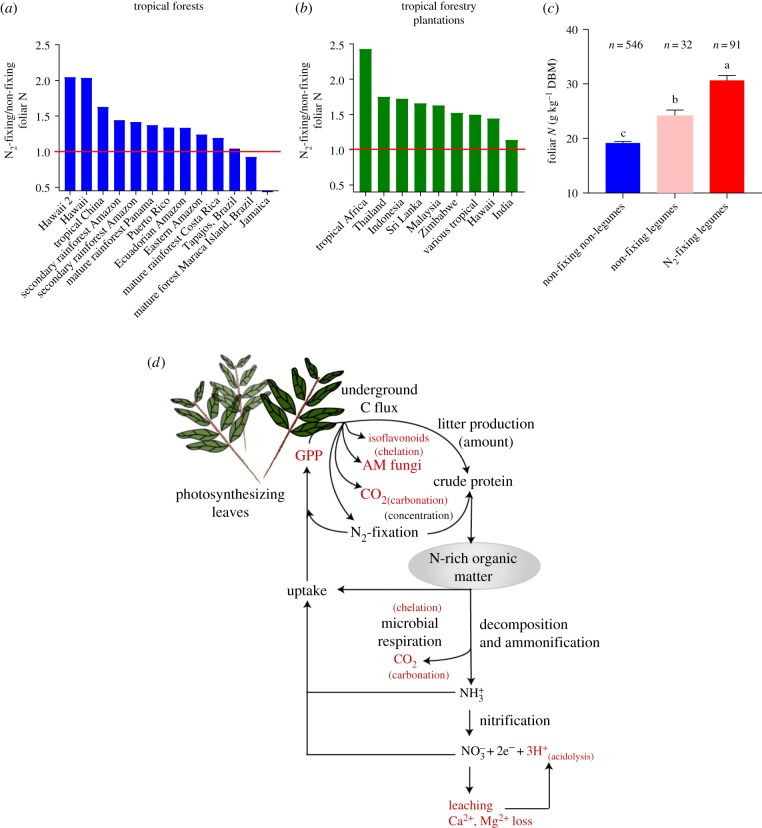
Foliar N ratios between N_2_-fixing and non-fixing non-legumes in (*a*) tropical forests, (*b*) tropical forestry plantations and (*c*) between the three functional groups and (*d*) pathways of the nitrogen-weathering feedback hypothesis. Red typeface depicts factors stimulating weathering with specific weathering reactions associated with those factors in brackets. In tropical forests, N_2_-fixing legumes exhibit an average of 34.58% (s.e.m. = 11.73%) higher leaf crude protein content than non-fixing tree species. In forestry plantations, N_2_-fixing legume species reveal on average 64.50% (s.e.m. = 11.57%) higher leaf crude protein content than non-fixing trees. Raw data and references are available in the electronic supplementary material. In (*c*), ‘*n*’ stands for number of species and DBM stands for dry biomass.

Second, N_2_-fixing legumes contain substantially higher leaf N than non-fixing tree species [36]. We performed a meta-analysis of 31 studies encompassing 561 tropical tree species (*n* = 680 measurements) to evaluate the N content of N_2_-fixing and non-fixing trees in natural forests and plantations across 22 different tropical regions (figure 2*a*,*b*). Our analysis shows that, despite considerable variation across sites, N_2_-fixers exhibit higher mean leaf N content than non-fixers (by 35% in natural tropical forests and by 65% in tropical forestry plantations) and non-fixing legumes (by 21%). These findings are consistent with a study of leaf N across Amazonian tropical forests that also reported N_2_-fixing legumes had higher leaf N content than both non-fixers as a whole and non-fixing legumes [37].

Third, this N-rich leaf tissue would cause increased input of N-rich compounds including proteins and amino acids to soils via litterfall. Such increased N input, in turn, would enrich soils in N and probably cause higher rates of productivity for non-fixing as well as N_2_-fixing trees. Evidence for such a major ecosystem impact comes from recent field studies: N_2_-fixing legumes provided approximately 50% of the N required for early growth of Panamanian secondary rainforests, supported rapid carbon accumulation in biomass of both fixers and non-fixers [38] and enhanced soil N [39] during periods of N limitation. Levels of N_2_-fixation in early Cenozoic fixers are hard to establish empirically but indirect evidence of greater insect damage from fossil leaves together with greater palatability and protein content of N_2_-fixing trees [13] support the assumption that ancient N_2_-fixers were capable of generating high N foliage.

We suggest these three characteristics of N_2_-fixing legumes probably entrain a suite of direct and indirect mechanisms that can enhance rates of rock weathering, as discussed below.

### (a) N_2_-fixing legume litter decomposition and microbial respiration

Litterfall and the decomposition of protein-enriched biomass would ultimately increase the flux of new fixed N into several linked soil processes (soil respiration, ammonification, nitrification) and pools (soil organic matter, dissolved organic N). The input of new N would trigger several weathering-related mechanisms (figure 2*d*).

First, the low C/N ratio of N_2_-fixing legume litter implies fast decomposition, greater microbial respiration and greater CO_2_ production than non-legume litter [40,41]. During decomposition, the majority of N-rich leaf tissue and its amino acids, amino sugars and other N-rich monomers will undergo ammonification and nitrification. Decomposition also generates organic acids and faster decomposition rates may facilitate passing the organic acid concentration threshold necessary to drive mineral weathering [42].

Second, N-rich organic matter can itself stimulate soil microbial activity and respiration. Although C inputs would have similar effects regardless of whether derived from decomposition of leguminous N-rich or non-leguminous N-poor litter, the lack of sufficient N can ultimately downregulate microbial respiration specifically under high CO_2_ regimes [43], such as those seen during the early Cenozoic (figure 1*b*). Addition of N_2_-fixing legume-derived N-rich litter may therefore have a dual function. First, it will fuel microbial respiration with the energy stored in the carbon–hydrogen (C–H) and carbon–carbon (C–C) bonds of its carbohydrate component. Second, because of its abundance in N and protein, it will promote microbial respiration by alleviating any existing N-limitation on microbial metabolism. *In situ* studies in tropical soils confirm augmented rates of microbial respiration in the combined glucose and N treatment compared with the glucose treatment alone [44].

Third, the dissolved CO_2_ generated by microbial respiration forms carbonic acid (H_2_CO_3_) which, in turn, acts as a major weathering agent [45] (electronic supplementary material, figure S1). Increased microbial respiration also positively correlates with the production of chelating organic acids, e.g. gluconic acid, a secreted by-product of microbial catabolism [46].

### (b) N_2_-fixing legume-driven soil acidification

Ammonia generated by ammonification during litter decomposition can undergo nitrification. In the process, each molecule of ammonia converted to nitrate generates three by-product H^+^ ions. Although these H^+^ ions are typically counterbalanced by plant secretion of anions (bicarbonate or organic acids) for each acquired 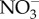, nitrate leaching can uncouple this relationship and promote the build-up of H^+^ in the soil. High levels of N_2_-fixation can exceed the rates at which N is immobilized within the system, resulting in enhanced 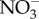 leaching (as discussed above) and enhanced transport of H^+^ to deeper soil horizons (where contents of unweathered minerals may be high). Tree ring data from tropical fossil woods indicate that climate seasonality was largely similar between early Cenozoic and modern tropical forests [47], supporting the view that nitrification patterns as affected by soil moisture/dryness [48] probably were comparable.

During the leaching of 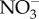 large amounts of counterbalancing cations (Ca^2+^, Mg^2+^, K^+^) released by cation exchange reactions with nitrification-generated H^+^ are leached too, resulting in the decline of soil cation exchange capacity and soil pH buffering capacities. This phenomenon has been recorded for N_2_-fixing forests of *Alnus rubra* in which large inputs of fixed N caused leaching, decreased cation concentration and increased soil acidification [49].

Despite the tight N budget of most tropical forest systems, substantial levels of nitrate leaching still occurs [33], suggesting that similar mechanisms probably operate in tropical forests rich in N_2_-fixing legumes. In addition, because of their N_2_-fixation, fixers tend to acquire lower relative amounts of negatively charged ions and produce larger organic acid loads per unit N resulting in the balancing H^+^ extrusion into the rhizosphere [50].

Consequently, pronounced soil acidification has been recorded in various N_2_-fixing species from herbs [50,51] to trees and shrubs of temperate forest [52,53] and tropical rainforest [39] areas. Recent analysis of tropical rainforests at four Neotropical locations revealed that forests rich in N_2_-fixers exhibited increased soil acidity (pH 4.1) and lower Ca^2+^ and Mg^2+^ concentrations than forests poor in N_2_-fixing legumes (pH 5.2) [54]. N_2_-fixing legume-driven acidification can promote weathering not only by acid attack (acidolysis) of the mineral lattice (electronic supplementary material, figure S1) but also by depleting soil cations through cation exchange, thus shifting the equilibrium towards further mineral dissolution.

### (c) N_2_-fixing legume-driven stimulation of net primary productivity

Ultimately, inorganic forms of fixed N are acquired from the soil solution by roots stimulating the N input into biomass, including that of neighbouring non-fixing trees. For instance, the non-fixing tropical trees *Peschiera*, *Psidium* [55], *Eucalyptus* [56] and *Terminalia* [57] all exhibited increased foliar N levels in N_2_-fixing legume-rich neighbourhoods compared with legume-poor settings. As foliar N correlates with increased levels of crude leaf protein, including the photosynthetic enzyme RUBISCO [58], the photosynthetic rates of individual trees and the net primary production (NPP) of such mixed fixer/non-fixer forests may be upregulated. Indeed, N_2_-fixing legumes exhibit up to twofold greater photosynthetic rates than the less N-rich leaves of non-fixing trees in Zimbabwe [59]. Similarly, non-fertilized mixed non-fixer/N_2_-fixer forestry plantations reveal augmented NPP rates compared with non-fixing forests in Brazil and Puerto Rico [60,61].

Fossil evidence supports N_2_-fixing legume-driven N-fertilization on productivity of tropical ecosystems. Presumed N_2_-fixing legume-dominated assemblages exhibited insect damage (linked to higher leaf N content) spread across fossil taxa relative to systems with fewer legumes in which foliar damage was more concentrated on legume leaves [13]. This observation indicates that as legume domination was established, N redistribution triggered by the input of N-rich litter increased N levels of neighbouring non-legumes (as observed in modern systems). The source of this N buffering effect is better explained by legumes capable of N_2_-fixation than non-fixing legumes because the patterns are consistent with the influx of new fixed N to the system.

Some canopy photosynthate from highly productive N_2_-fixing legume-rich forests will be allocated to symbiotic mycorrhizal fungi. The mycelial networks of these fungi grow in intimate contact with mineral grains, thus driving enhanced rock weathering and inorganic nutrient release via chelation, carbonation and acidolysis (electronic supplementary material, figure S1) [45]. Greater gross primary production (GPP) and its related NPP rates also correlate with greater root respiration (with associated production of carbonic acid) and organic acid leaching, which promotes further weathering [45] (electronic supplementary material, figure S1). N_2_-fixing legume-enhanced forest NPP can also increase the demand for nutrients and thus further necessitate more extensive soil exploration via roots and mycorrhizal fungi, and eventually enhanced rock weathering. Therefore, increased N inputs could indirectly increase rock weathering via stimulation of rainforest NPP in legume-rich communities compared with *Nypa* and other Late Cretaceous palm forests as well as to legume-poor early Cenozoic analogues.

### (d) Accessory mechanisms of N_2_-fixing legume-driven weathering

The unique ability of legumes (including many rainforest N_2_-fixing legume trees [62,63]) to synthesize and exude isoflavonoids [64] may also impact weathering rates. Isoflavonoids enhance phosphorus (P) and iron (Fe) solubilization from the mineral vivianite by acting as soil chelators (electronic supplementary material, figure S1) as well as by decreasing organic acid decomposition [65]. Comparison between the estimated low-molecular organic acid exudation by lowland tropical rainforest trees (approx. 25 µg C g^−1^ dry biomass (DBM) root h^−1^) [66] and isoflavonoid exudation of the N_2_-fixer *Lupinus albus* (approx. 31 µg C g^−1^ DBM root h^−1^) [67] (see the electronic supplementary material for detailed calculations) suggests that isoflavonoids could contribute to the pool of plant-derived chelating agents in legume-rich forest soils.

Isoflavonoids are crucial in establishing the N_2_-fixing legume-rhizobial symbiosis by enabling both attraction and priming of rhizobial partners [68]. They attract larger soil rhizobial populations [69] of nodulation-competent strains of *Burkholderia, Rhizobium* and *Mesorhizobium—*members of all of these genera have been shown to exert strong chelating activities [70]. Soil pH, C, N and C/N ratio are also important determinants of microbial community structure [71]. Finally, legume-mediated changes in soil chemistry may change microbial community of the mineralosphere selecting for nitrophilic and acidophilic bacterial taxa.

## 4. N_2_-fixing legume-rich forest responses to a CO_2_-rich early Cenozoic atmosphere

**Figure 3.**
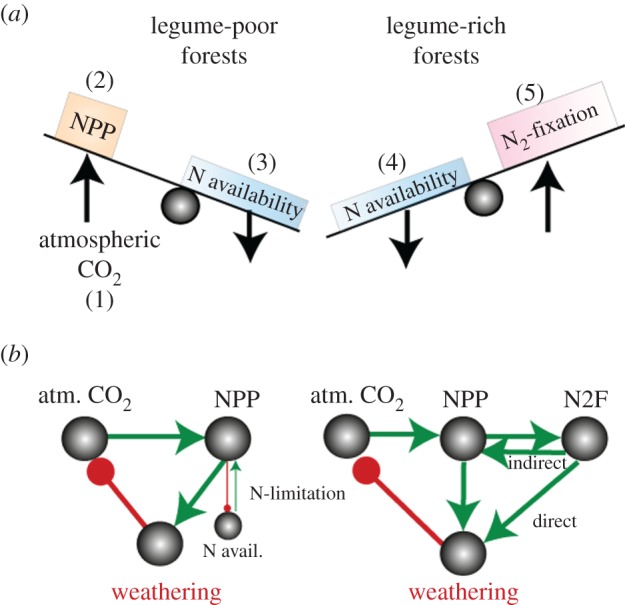
Atmospheric CO_2_, NPP, weathering and N feedbacks. (*a*) Ecosystem effects of elevated CO_2_ levels in legume-poor and rich forests; (*b*) differences in feedback relationships between rich and poor forests. In both forest types, high atmospheric CO_2_ levels (1) promote a proportional NPP increase (2) which transitions the system to low N-availability (3). Ultimately, in poor forests that would result in a negative feedback on NPP. In rich forests, however, low N-availability (3) can upregulate N_2_-fixation rates and recruitment of N_2_-fixers (4) thus alleviating N limitations and allowing for an unchanged CO_2_-NPP relationship. Green arrows indicate positive relationships, whereas red ball-ending lines—negative relationships; N_2_F, N_2_-fixation.

The rise of N_2_-fixing legume-rich tropical forests during the early Cenozoic coincides with elevated atmospheric CO_2_ concentrations, with potential feedbacks on primary production and weathering (figure 1*b*, figure 3). Evidence for the mechanisms that may govern this potential feedback comes from free air CO_2_-enrichment (FACE) experiments. In the Oak Ridge, TN, USA, FACE experiment, the non-fixing AM *Liquidambar styraciflua* trees showed a 24% increase in NPP during the first 6 years of exposure to elevated CO_2_ [72]. However, over the next 5 years the positive CO_2_-enrichment effect decreased to +9% in 11-year old stands as ecosystem N stocks declined [72], suggesting progressive soil N-limitation on tree NPP in the long-term under high CO_2_ [72,73]. N_2_-fixing legumes may mitigate this N-limitation mechanism under a high CO_2_ atmosphere because N-limitation would favour recruitment of N_2_-fixing legumes and/or upregulate their fixation rates [74,75]. Fossil evidence suggests that N_2_-fixing legumes may increase in abundance under such conditions. During the transient climate warming event across the Palaeocene–Eocene thermal maximum (PETM; 55.8 Ma) that is linked to a rise in atmospheric CO_2_ and continental weathering regimes [76], the abundance of fossilized leguminous leaf specimens increased to 73% and then declined to 21% post-PETM in the Bighorn Basin, USA [23]. Further evidence from PETM sites dominated by legumes corroborates extensive N_2_-fixation capacity increasing N availability to the system (as discussed above) [13].

Physiologically, elevated CO_2_ can promote nodulation and N_2_-fixation [77–79], mycorrhization [80] and photosynthetic rates, and therefore may allow N_2_-fixing legume productivity to increase proportionally more in response to CO_2_ than non-legumes [77,79]. Furthermore, nodules represent additional sinks exchanging the increased flux of assimilates for fixed N thus curtailing the photosynthetic acclimation to elevated CO_2_ when unconstrained by other factors [81], allowing higher photosynthetic rates to persist. Those effects could promote N_2_-fixer recruitment, upregulated N_2_-fixation rates and greater dominance at high CO_2_ concentrations [82]. A FACE experiment at Oak Ridge analysed the CO_2_ response of over 2000 seedlings from 14 different temperate tree species. After 5 years, the N_2_-fixing legume *Robinia pseudoacacia* exhibited an order of magnitude higher biomass response than all of the non-fixing angiosperm trees [83]. Controlled environment pot-based CO_2_-enrichment experiments indicate that the photosynthesis and growth responses of nodulated N_2_-fixing Leguminosae rainforest trees were significantly greater than that of non-leguminous species investigated [84]. Although there are clear limitations in extrapolating from these studies to legumes of early Cenozoic tropical forests, the mechanistic basis of the CO_2_ response—linked to alleviation of N-limitation—would still hold.

Based on these findings, we conceptualize that different feedback loops operated between non-legume and N_2_-fixing legume forests, atmospheric CO_2_ and climate in the Cenozoic (figure 3). In *non-fixing forests* like those that existed prior to legume evolution or in legume-poor tropical forests of the early Cenozoic, increased atmospheric CO_2_ would stimulate NPP until available soil resources—probably N and P in many locations—are exhausted (figure 3*a*: feedbacks 1-2-3). Progressive N-limitation could therefore uncouple the ‘standard’ relationship between NPP, CO_2_ and weathering [85] in legume poor forests (figure 3*b*). By contrast, however, in *legume-rich forests,* progressive N-limitation would probably further promote recruitment of N_2_-fixers and the up-regulation of N_2_-fixation rates (figure 3*a*: feedbacks 1-2-3-4-5), as observed in modern N-limited rainforests [38]. This could allow NPP to respond to increasing CO_2_ and help promote continued weathering (figure 3*b*). Additionally, biological weathering processes are strengthened by inputs of N-rich legume litter and associated downstream processes. Combined, this evidence indicates that in CO_2_-rich conditions, the significant role of legumes in maintaining enhanced weathering regimes in early tropical forests may be amplified.

## 5. Evolutionary drivers of enhanced weathering by N_2_-fixing legumes

Central to our feedback analyses (figure 3) is the idea that N_2_-fixing legumes are associated with higher weathering rates than non-legume trees. This effect, in turn, may have evolved in response to a disproportionately high demand for P, molybdenum (Mo) and Fe across legume taxa. P and Mo have been identified as potentially limiting factors of N_2_-fixation within tropical forests [86–88]. These limitations may occur because the most common type of nitrogenases involved in symbiotic N_2_-fixation requires an Fe/Mo complex acting as a cofactor [86] while high P intake accommodates for enhanced production of energy-rich metabolites (e.g. ATP) and membranes during nodule organogenesis [89]. Linked to the probable greater P demand driven by higher rates of growth, some but not all N_2_-fixing legumes may have higher foliar P levels than non-fixing trees (electronic supplementary material, table S1). Fe is also required for production of leghaemoglobin in nodules for oxygen binding [90]. Fe is very abundant in tropical soils but it is highly insoluble. Most P in soils is also insoluble in complexes with aluminium (Al)- and Fe-bearing secondary minerals, and fresh Mo and P inputs originate from weathering of otherwise plant-unavailable mineral sources. Both the dissolution of insoluble P and Fe and the release of mineral-bound Mo rely upon the same weathering mechanisms that include chelation and acidolysis [91] (electronic supplementary material, figure S1). Al and iron phosphate minerals such as variscite and vivianite, respectively, dissolve faster at pH < 6, a process exacerbated by organic acids [91,92].

Overall, the processes of N_2_-fixation and nodule formation require an array of sparingly soluble (P, Fe) or scarce soil minerals (Mo). This observation suggests that the mechanisms of enhanced weathering overlap with those driving acquisition of elements essential for N_2_-fixing legumes. It provides a mechanism that would promote the evolution of adaptive strategies in tropical legumes leading to enhanced weathering and thereby unlocking sparingly soluble limiting nutrients. Our hypothesized mechanisms that relate N_2_-fixing legume functioning to weathering rates are suitable for direct investigation in the field and laboratory, and future studies will hopefully further elucidate the relative importance of each of the mechanisms of the hereby proposed hypothesis.

## 6. Conclusion

Fossils and molecular dating suggest that a worldwide shift from palm-dominated communities to ‘modern’ tropical forests occurred early in the Cenozoic and involved the development of N_2_-fixing legume-rich and symbiotically diverse communities. Based on our analyses of potential effects on forest ecosystem biogeochemical C and N cycling, we propose that the increasing abundance of N_2_-fixing legumes in tropical forests amplified weathering rates through several interconnected pathways. Firstly, N_2_-fixing legumes increased soil inputs of N-rich organic matter (by an estimated 35–65% based on modern analogues) which can promote microbial respiration and carbonation as well as progressive soil acidification resulting from leaching and compensatory H^+^ extrusion. Subsequently, increased N inputs may have fuelled greater N-availability stimulating forest NPP, thus driving further carbonation, organic acid chelation and rhizospheric weathering activities. Lastly, exudation of N-costly isoflavonoids unique to legumes could have provided an additional source of chelating activities that cause rock weathering. Together with soil acidification and decreasing C/N ratios these effects could have indirectly driven shifts in the weathering potential of the soil microbial community.

We suggest the global evolution of tropical forests rich in N_2_-fixing legumes in the early Cenozoic in concert with abiotic drivers, including reduced subduction of oceanic crust and the rise of the Himalayas/Tibetan plateau [32,93], could have contributed to regimes of enhanced weathering over pantropical areas with consequent feedbacks on global climate. Furthermore, N_2_-fixing legumes help maintain the NPP response to atmospheric CO_2_ concentration. In an evolutionary context, tropical N_2_-fixing legumes appear to enhance rock weathering as a possible adaptation to unlock previously unavailable P, Mo and Fe mineral sources, thus alleviating limitations on N_2_-fixation processes.

## Supplementary Material

Supplementary Figures 1 and 2

## Supplementary Material

Supplementary Table 1

## Supplementary Material

Organic Acid Exudation Rates.

## Data accessibility

This article has no additional data.
