# Supplementary material for: N2-fixing tropical legume evolution: a contributor to enhanced weathering through the Cenozoic?
Source: Proc Biol Sci. 2017 Aug 16;284(1860):20170370. doi: 10.1098/rspb.2017.0370 (PMC5563791; doi:10.1098/rspb.2017.0370)
Supplement: Supplementary Figures 1 and 2 [file rspb20170370supp1.docx]

**Supplementary Material for**

**N_2_-fixing tropical legume evolution: a contributor to enhanced weathering through the Cenozoic?**

Dimitar Z. Epihov, Sarah A. Batterman, Lars O. Hedin, Jonathan R. Leake, Lisa M. Smith and David J. Beerling

**Supplementary Figure 1. Illustrated glossary of weathering reactions**

The following processes are often enhanced by biological (biotic) systems such as root exudation, microbial physiology, soil respiration and organic matter decomposition and leaching and therefore represent the basis of biological weathering [1].

***Chelation (acido-complexolysis; complexation)*** – the reaction of complexing between metal ions from minerals/rocks with organic molecules (chelating agents; chelators) via the formation of coordination bonds. Important in biological weathering. Organic acids such as citric acid, oxalic acid, tartaric acid, acetic acid, lactic acid, gluconic acid and amino acids are major chelating agents. Chelating organic acids may also produce protons during their dissociation which can further attack minerals through acidolysis which is why some sources refer to chelation also as acido-complexolysis [2].

*Suggested example:* Oxalic acid + olivine 🡪 [Mg^2+^ : oxalate] complex + weathered olivine + 2H^+^

**
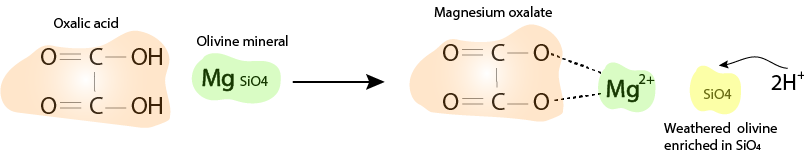
**

***Carbonation* –** CO_2_ in soil produced by biological activity such as respiration often dissolves in water forming the weak carbonic acid (H_2_CO_3_). Carbonic acid can react with silicate minerals producing metal carbonates. The process can be sped-up by the presence of the enzyme carbonic anhydrase [3].

*Suggested example:*


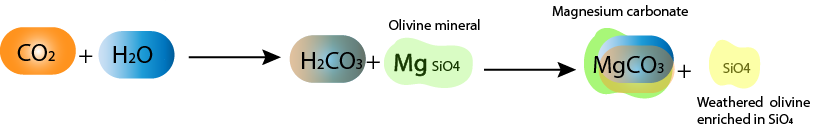


***Acidolysis*** ***(simple acidolysis, acid attack, protonation)*** – a process in which protons (generated biologically or by acid dissociation) replace the metal cations from mineral surfaces and bring the mineral metals to solution.

*Suggested example:*

**Figure I. Basalt boulders in the tropical forests of Barro Colorado Island,**

**Panama –** the presence of boulders (here seen with weathered surface of

altered colour) may indicate that high amounts of volcanic mineral materials at

different stages of weathering are contained within the soil horizons where chelation,

acidolysis and carbonation driven by forest processes can stimulate their dissolution.

Photo credit: Dimitar Z. Epihov.


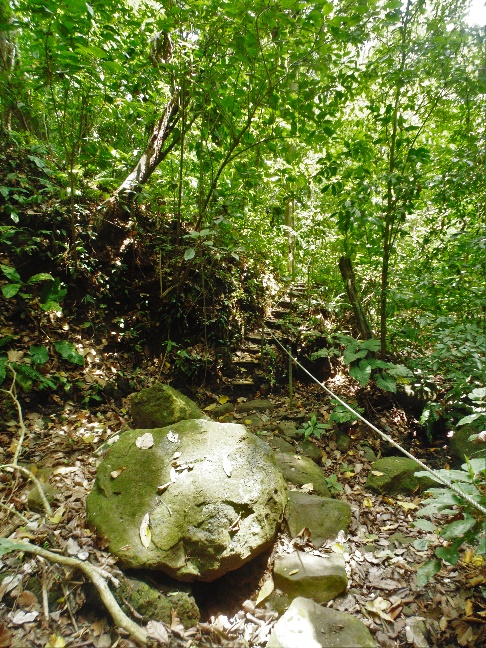


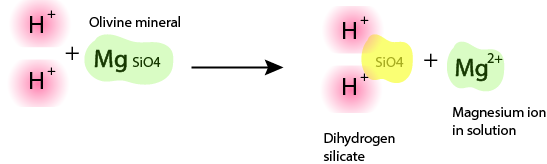


**Supplementary Figure 2. Functional symbiotic diversity within Leguminosae – coupling extant and fossil evidence**

Root microbial symbioses can be divided into 2 major groups - dipartite (that is symbioses between a plant host and a single symbiont group) and multipartite (that is symbioses between a plant host and two or more symbiotic partners). Plants with dipartite symbioses include arbuscular mycorrhizal (AM) plants, ectomycorrhizal (EM), ericoid mycorrhizal (ERM) etc. Plants with multipartite symbioses are the group of N_2_-fixing and arbuscular mycorrhizal plants (NAM), N_2_-fixing and ectomycorrhizal plants (NEM), and N_2_-fixing dual arbuscular-ectomycorrhizal plants (NAEM).

Leguminosae is one of the most symbiotically-rich plant families with members known to exhibit AM, EM, NAM, NEM [4],[5] or NAEM [6] properties. Symbiotic assignment to fossil taxa is based upon the symbiotic characteristics of extant members of that taxon as found in the Supplementary curated by Werner *et al*. in [7] (except *Xylia* - [8] and *Maniltoa* - [9]). Lists of ectomycorrhizal legumes are found in [10] and [11].

| **Functional type** |  | **Palaeocene/Eocene Fossil Legume Taxa** |
| --- | --- | --- |
| ***NAM*** | **Figure I.** **N_2_-fixing AM legumes of Neotropical rainforests: *Inga cocleensis –* an extant member of the ancient *Inga* genus with origins in the early Cenozoic.** Shown here are flowers and leaves, canopy, *Burkholderia* root nodules and Trypan Blue-stained intraradical AM hyphae. Photo credit: Dimitar Z. Epihov | *Inga* [12]*,*[13]*,*[14]*, Prosopis* [15]*,*[16]*, Mimosa* [15]*, Chamaechrista* [12]*, Acacia* [13]*,*[16]*,*[17]*,*[18]*,*[19]*, Swartzia* [16]*,*[20]*, Albizia* [15]*,*[16]*, Penthaclethra* [15]*,*[19]*, Adentanthera* [15]*,*[19] *Ormosia* [16]*, Sophora* [13]*,*[16]*, Robinia* [16]*,*[20]*, Diplotropis* [16]*, Canavalia* [13]*, Dalbergia* [13]*,*[20]*, Machaerium* [16]*, Strongydolon* [21]*, Pongamia* [21]*, Neptunia* [16]*, Derris* [22]*, Desmodium* [22] *, Millettia* [22]*, Maniltoa* [23]*, Crudia* [23]*,*[24]*, Xylia* [16] |
| ***EM*** |  | *Aphanocalyx* [17]*, Afzelioxylon/Afzelia* [25]*,*[26]*, Brachystegia* [27]*,*[28]*, Julbernardia* [29] |
| ***AM*** |  | *Ablygonocarpus* [15]*, Gymnocladus* [20]*, Cladrastis* [20], *Senna* [16], *Calpocalyx* [15], *Cassia* [30], *Cynometra* [31], *Peltogyne* [16], *Bauhinia* [20]*, Vouapa* [21]*, Hymenaea* [23]*, Caesalpinia* [16] |

**Modern analogues of Cenozoic fossil forests**. The occurrence of fossils of the above taxa at different sites suggests early Cenozoic forests exhibited compositional patterns analogous to major types of modern forests with Leguminosae as an important family in both species-rich NAM forests and monodominant forests of EM or AM legumes. We suggest fossils of *Inga, Swartzia, Machaerium* and the AM legume taxa (*Senna, Cassia*) might be analogous to species-rich NAM legume tropical rainforests of modern Amazon, Panama and Costa Rica [32]. Fossils of *Brachystegia* and *Julbernardia* are suggestive of monodominant EM dry tropical forests like miombo woodlands in Africa [33]. Abundant *Acacia* fossil records are often interpreted as dry tropical forests analogous to savanna *Acacia* woodland communities currently found in Africa and Mexico [34]. Fossil assemblages of *Cynometra*- and pollen of *Peltogyne*-affinity might be analogous to the monodominant legume AM communities forming *Cynometra alexandrii* forests in Africa and that of *Peltogyne gracilipes* in Amazon [35]. Finally, we suggest that the presence of fossil EM *Aphanocalyx* and *Afzelia* might be indicative as accessory EM species found in modern monodominant EM rainforests like the EM legume *Microberlinia bisulcata* monodominant forests in Cameroon [11],[36].


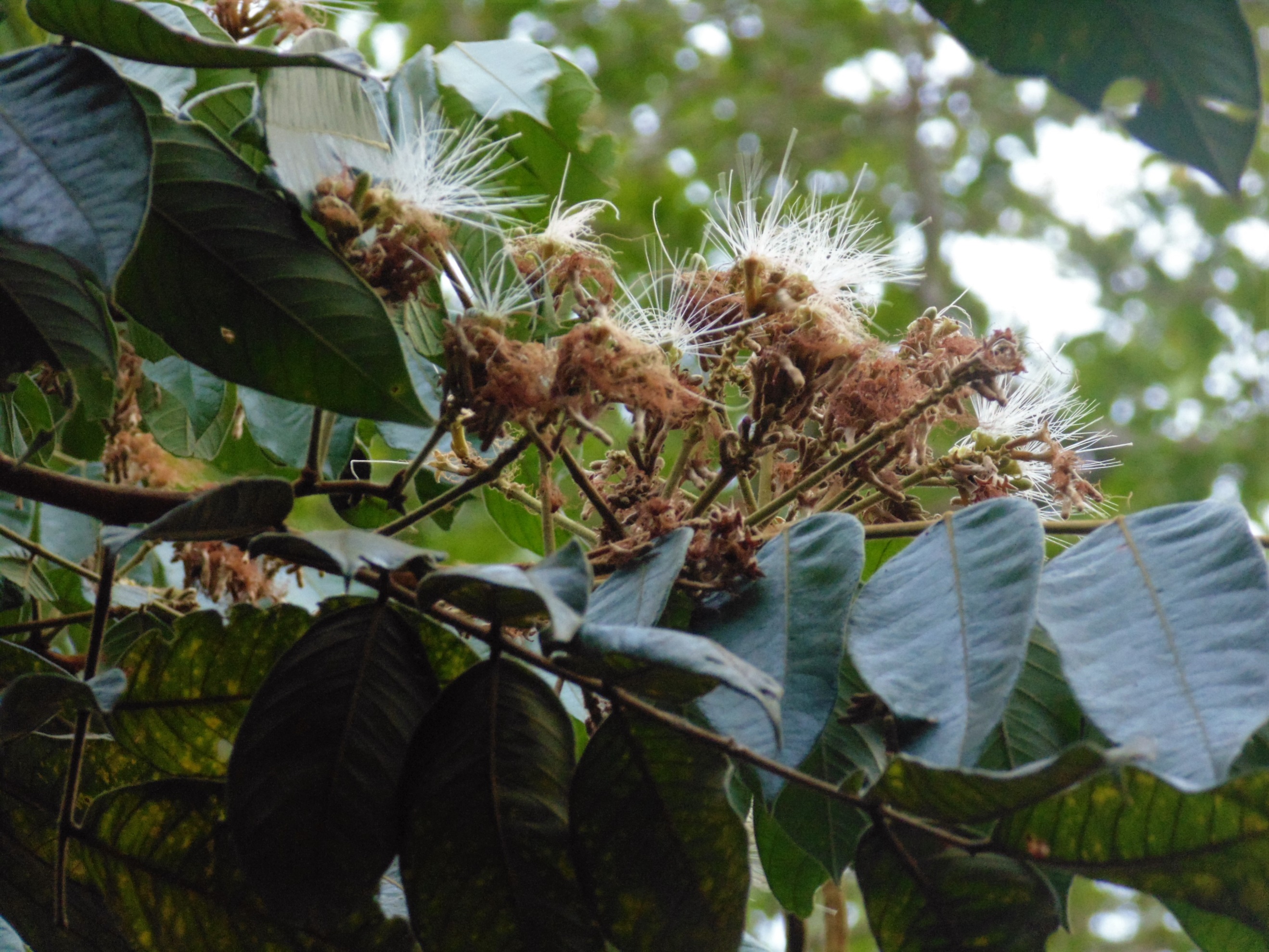

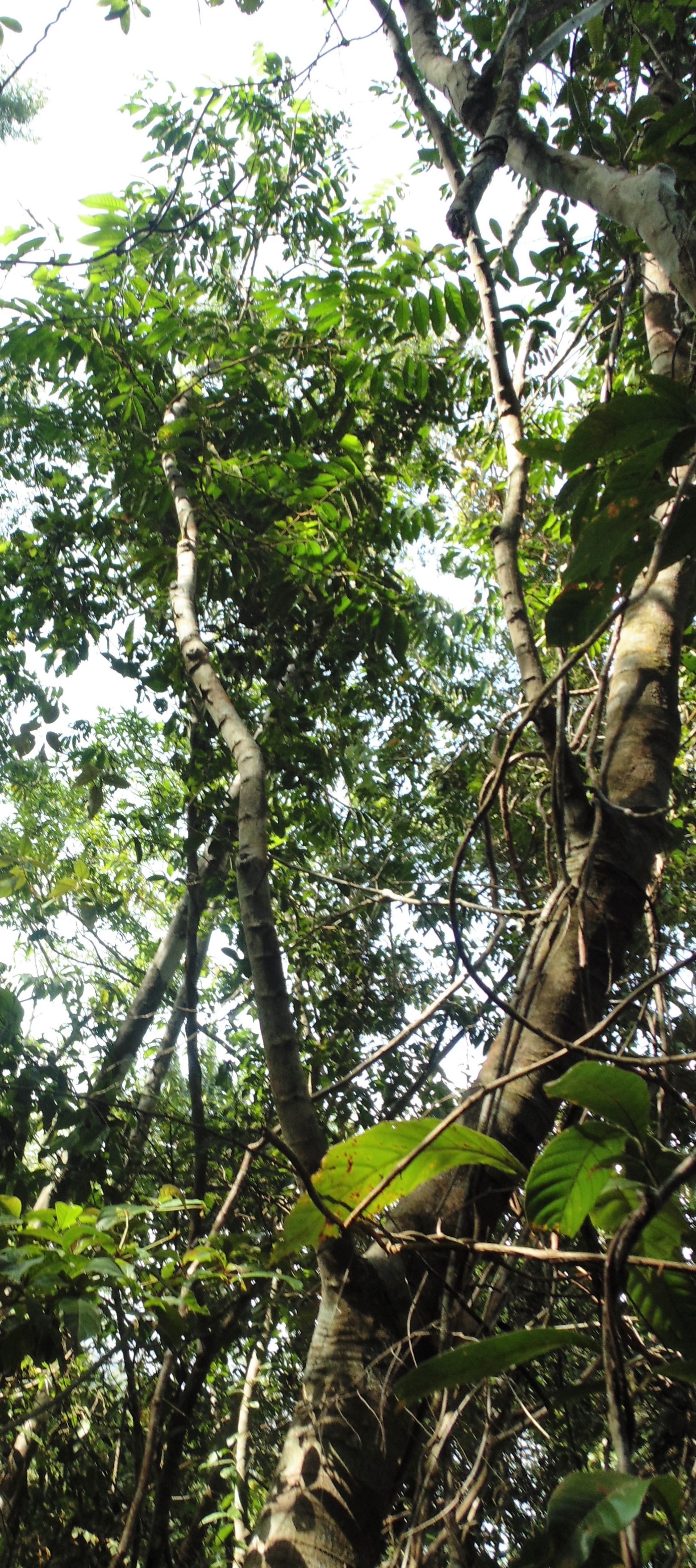


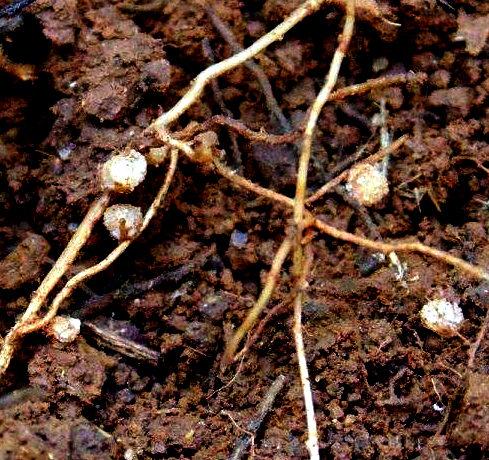

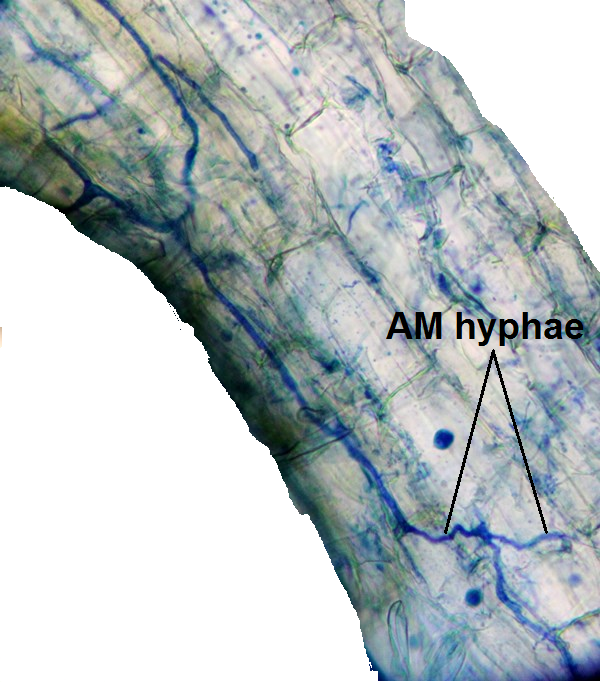


**Supplementary References**

**Supplementary References**

1. Taylor, L. L., Leake, J. R., Quirk, J., Hardy, K., Banwart, S. a. & Beerling, D. J. 2009 Biological weathering and the long-term carbon cycle: Integrating mycorrhizal evolution and function into the current paradigm. *Geobiology* **7**, 171–191. (doi:10.1111/j.1472-4669.2009.00194.x)

2. Van Rompaey, K., Van Ranst, E., Verdoodt, A. & De Coninck, F. 2007 Use of the test-mineral technique to distinguish simple acidolysis from acido-complexolysis in a Podzol profile. *Geoderma* **137**, 293–299. (doi:10.1016/j.geoderma.2006.08.014)

3. Li, W., Yu, L. J., He, Q. F., Wu, Y., Yuan, D. X. & Cao, J. H. 2005 Effects of microbes and their carbonic anhydrase on Ca2+ and Mg2+ migration in column-built leached soil-limestone karst systems. *Appl. Soil Ecol.* **29**, 274–281. (doi:10.1016/j.apsoil.2004.12.001)

4. Hogberg, P. 1986 Nitrogen-Fixation and Nutrient Relations in Savanna Woodland Trees (Tanzania). *J. Appl. Ecol.* **23**, 675–688. (doi:10.2307/2404045)

5. Diédhiou, a. G., Guèye, O., Diabaté, M., Prin, Y., Duponnois, R., Dreyfus, B. & Bâ, a. M. 2005 Contrasting responses to ectomycorrhizal inoculation in seedlings of six tropical African tree species. *Mycorrhiza* **16**, 11–17. (doi:10.1007/s00572-005-0007-8)

6. Hopkins, M. S., Reddell, P., Hewett, R. K. & Graham, a W. 1996 Comparison of root and mycorrhizal characteristics in primary and secondary rainforest on a metamorphic soil in north Queensland, Australia. *J. Trop. Ecol.* **12**, 871–885. (doi:10.1017/S0266467400010130)

7. Werner, G. D. a, Cornwell, W. K., Sprent, J. I., Kattge, J. & Kiers, E. T. 2014 A single evolutionary innovation drives the deep evolution of symbiotic N2-fixation in angiosperms. *Nat. Commun.* **5**, 4087. (doi:10.1038/ncomms5087)

8. Teamroong, N. & Boonkerd, N. 2006 Rhizobial Production Technology. In *Microbial Biotechnology in Agriculture and Aquaculture vol. 2* (ed R. Ray), pp. 77–110. Science Publishers.

9. Lewin, A. et al. 1987 Multiple host-specificity loci of the broad host-range Rhizobium sp. NGR234 selected using the widely compatible legume Vigna unguiculata. *Plant Mol. Biol.* **8**, 447–459. (doi:10.1007/BF00017990)

10. Alexander, B. Y. I. J. & Hogberg, P. 1986 Ectomycorrhizas of Tropical Angiospermous Trees. *New Phytol.* **102**, 541–549. (doi:10.1111/j.1469-8137.1986.tb00830.x)

11. Newbery, D. & Alexander, I. 1988 Ectomycorrhizal rain‐forest legumes and soil phosphorus in Korup National Park, Cameroon. *New Phytol.* **109**, 433–450.

12. Calvillo-Canadell, L. & Cevallos-Ferriz, S. R. S. 2005 Diverse Assemblage of Eocene and Oligocene Leguminosae from Mexico. *Int. J. Plant Sci.* **166**, 671–692.

13. Berry, E. W. 1930 Revision of the Lower Eocene Wilcox flora of the southeastern states: With descriptions of new species, chiefly from Tennessee and Kentucky. *US Gov. Print. Off.* **156**.

14. Irving, R. S. & Stuessy, T. F. 1971 A New Paratropical Angiosperm Florule in the Eocene Rockdale Formation of Bastrop County, Texas. *Southwest. Nat.* **16**, 111–116.

15. Caccavari, M. A. 1996 Analysis of the South American fossil pollen record of Mimosoideae (Leguminosae). *Rev. Palaeobot. Palynol.* **94**, 123–135.

16. Herendeen, P. S., Crepet, W. L. & Dilcher, D. L. 1992 The fossil history of the Leguminosae: phylogenetic and biogeographic implications. In *Advances in Legume Systematics Part 4. The Fossil Record*, pp. 303–316. Kew Publishing.

17. Herendeen, P. S. & Jacobs, B. F. 2000 Fossil Legumes from the Middle Eocene (46.0 Ma) Mahenge Flora of Singida, Tanzania. *Am. J. Bot.* **87**, 1358–1366.

18. Wang, Q., Ferguson, D. K., Feng, G.-P., Ablaev, A. G., Wang, Y.-F., Yang, J., Li, Y.-L. & Li, C.-S. 2010 Climatic change during the Palaeocene to Eocene based on fossil plants from Fushun, China. *Palaeogeogr. Palaeoclimatol. Palaeoecol.* **295**, 323–331. (doi:10.1016/j.palaeo.2010.06.010)

19. Maley, J. 1996 The African rain forest – main characteristics of changes in vegetation and climate from the Upper Cretaceous to the Quaternary. *Proc. R. Soc. Edinburgh. Sect. B. Biol. Sci.* **104**, 31–73. (doi:10.1017/S0269727000006114)

20. Taylor, D. W. 1990 Paleobiogeographic relationships of angiosperms from the Cretaceous and early Tertiary of the North American area. *Bot. Rev.* **56**, 279–417. (doi:10.1007/BF02995927)

21. MacGinitie, H. D. 1941 H. D. MacGinitie. 1941. A Middle Eocene Flora from the Central Sierra Nevada. 534: 1-178. *Carnegie Inst. Washingt. Publ.* **534**, 1–178.

22. Awasthi, N. 1992 Indian fossil legumes. In *Advances in Legume Systematics 4: The Fossil Record*, pp. 225–250.

23. Graham, A. 1992 The current status of the legume fossil record in the Carribean region. In *Advances in Legume Systematics 4: The Fossil Record*, pp. 161–167.

24. Herendeen, P. S. & Crane, P. R. 1992 Early Caesalpinioid fruits from the Palaeogene of Southern England. In *Advances in Legume Systematics 4: The Fossil Record*,

25. Pan, A. D., Jacobs, B. F. & Herendeen, P. S. 2010 Detarieae sensu lato (Fabaceae) from the Late Oligocene (27.23 Ma) Guang River flora of north-western Ethiopia. *Bot. J. Linn. Soc.* **163**, 44–54. (doi:10.1111/j.1095-8339.2010.01044.x)

26. In press. Afzelioxylon furoni (N° inventaire 5731) http://albinoni.snv.jussieu.fr/DB/specimens/fiche/spe442.html.

27. Shakryl, A. K. 1992 Leguminosae species from the Tertiary of Abkhazia. In *Advances in Legume Systematics: Part 4. The Fossil Record* (eds P. S. Herendeen & D. L. Dilcher), Kew Publishing.

28. Ettingshausen, C. B. 1879 Report on Phyto-Palaeontological Investigations of the Fossil Flora of Sheppey. *Proc. R. Soc. London* **29**, 388–396. (doi:10.1098/rspl.1879.0065)

29. Postigo Mijarra, J. M., Barrón, E., Gómez Manzaneque, F. & Morla, C. 2009 Floristic changes in the Iberian Peninsula and Balearic Islands (south-west Europe) during the cenozoic. *J. Biogeogr.* **36**, 2025–2043. (doi:10.1111/j.1365-2699.2009.02142.x)

30. Wilf, P., Johnson, K. R., Cúneo, N. R., Smith, M. E., Singer, B. S. & Gandolfo, M. a 2005 Eocene plant diversity at Laguna del Hunco and Río Pichileufú, Patagonia, Argentina. *Am. Nat.* **165**, 634–650. (doi:10.1086/430055)

31. Cantrill, D. J., Bamford, M. K., Wagstaff, B. E. & Sauquet, H. 2013 Early Eocene fossil plants from the Mwadui kimberlite pipe, Tanzania. *Rev. Palaeobot. Palynol.* **196**, 19–35. (doi:10.1016/j.revpalbo.2013.04.002)

32. Batterman, S. A. 2013 Symbiotic N2 fixation in tropical forests: Scaling from individuals to ecosystems.

33. Malmer, a 2007 General ecological features of miombo woodlands and considerations for utilization and management. *MITMIOMBO--Management Indig. Tree Species Ecosyst. Restor. Wood Prod. Semi-Arid Miombo Woodlands East. Africa. Proc. First MITMIOMBO Proj. Work. held Morogoro, Tanzania* , 6–12.

34. Greenberg, R., Bichier, P. & Sterling, J. 1997 Acacia, cattle and migratory birds in southeastern Mexico. *Biol. Conserv.* **80**, 235–247. (doi:10.1016/S0006-3207(96)00137-1)

35. Torti, S. D. & Coley, P. D. 1999 Tropical Monodominance: A preliminary test of the Ectomycorrhizal Hypothesis. *Biotropica* **31**, 220–228.

36. Esoeyang, E., Bechem, T., Chuyong, G. B. & Fon, B. T. 2014 A survey of mycorrhizal colonization in the 50-ha Korup Forest Dynamic Plot in Cameroon. *Am. J. Plant Sci.* **5**, 1403–1415.
