# Supplementary material for: N2-fixing tropical legume evolution: a contributor to enhanced weathering through the Cenozoic?
Source: Proc Biol Sci. 2017 Aug 16;284(1860):20170370. doi: 10.1098/rspb.2017.0370 (PMC5563791; doi:10.1098/rspb.2017.0370)
Supplement: Organic Acid Exudation Rates. [file rspb20170370supp3.docx]

**Supplementary Note**

**Calculation of exudation rates**

1) Organic acid (OA) exudation

The rate of low-weight organic acid exudation was taken from [1] reporting rates of three types of rainforest – lowland P-rich, montane P-poor, montane P-rich. However, although highest legume abundance is found in lowland forests [2], most tropical forests are P-poor so we used the exudation rate of montane P-poor forests as a proxy for rainforest tree exudation rates. Conversions below:

16.6% of aboveground net primary productivity (ANPP) allocated to organic acid exudation in P-poor tropical forests [1]

Lowland P-limited rainforest systems ANPP = 4.25 Mg C ha^-1^ yr^-1^ [3]

- exudation will be 0.166x4.25 = 0.7055 Mg C ha^-1^ yr^-1^ = 0.00007055 Mg C m^-2^ yr^-1^ = 70.55 g C m^-2^ yr^-1^ = 0.008165 g C m^-2^ h^-1^

The average biomass of living fine roots (which can exude organic acids) in tropical evergreen rainforests is 0.33 kg m^-2^ [4]

- Low-weight OA exudation rate will be 0.008165/330 = 2.474x10-5 g C g^-1^ DW root h^-1^ =

= **24.74 µg C g^-1^ DW root h^-1^**

2) Isoflavonoid exudation in the N_2_-fixing model plant for exudation studies – white lupine (*Lupinus albus*)

The rate of isoflavonoid exudation is taken from [5] reporting rates of *Lupinus albus* cluster and non-cluster roots. As cluster roots are considered a type of rhizomorphic specialization, we used the exudation rates of non-cluster roots instead as a more general scenario for root morphology.

The combined exudation rate of the 4 major isoflavonoids is as follows:

1 (genistein 7-*O*-diglucoside) + 6 (genistein 6’-*O*-malonyl-diglucoside) + 4 (genistein) =

= 11 µg g^-1^ FW root h^-1^

However in order to calculate that as µm C g^-1^ FW root h^-1^, we used the percentage C % (m/m) in each of those compounds which are as follows: 55%, 52%, and 67%

- isoflavonoid exudation rate = (0.55x1) + (0.52x6) + (0.67*4) = 0.55 + 3.12 + 2.68

= 6.35 µg C g^-1^ FW root h^-1^

That is measured in g FW (Fresh Weight) root. To convert that into g DW (Dry Weight) we used the FW root/DW root ratio for lupine provided in [6] which averages 4.94/1.

- Isoflavonoid exudation rate = 6.35*4.94 = **31.37 µg C g^-1^ DW root h^-1^**

**Supplementary References:**

1 Aoki, M. *et al.* (2012) Environmental Control of Root Exudation of Low-Molecular Weight Organic Acids in Tropical Rainforests. *Ecosystems* 15, 1194–1203

2 ter Steege, H. *et al.* (2006) Continental-scale patterns of canopy tree composition and function across Amazonia. *Nature* 443, 444–447

3 Aragão, L.E.O.C. *et al.* (2009) Above- and below-ground net primary productivity across ten Amazonian forests on contrasting soils. *Biogeosciences* 6, 2441–2488

4 Jackson, R.B. *et al.* (1997) A global budget for fine root biomass, surface area, and nutrient contents. *Proc. Natl. Acad. Sci. U. S. A.* 94, 7362–7366

5 Weisskopf, L. *et al.* (2006) Isoflavonoid exudation from white lupin roots is influenced by phosphate supply , root type and cluster-root stage. *New Phytol.* 171, 657–668

6 Sprent, J.I. (1973) Growth and Nitrogen fixation in Lupinus arboreus as affected by shading and water supply. *New Phytol.* 72, 1005–1022
